# Transcriptional profiling of bovine milk using RNA sequencing

**DOI:** 10.1186/1471-2164-13-45

**Published:** 2012-01-25

**Authors:** Saumya Wickramasinghe, Gonzalo Rincon, Alma Islas-Trejo, Juan F Medrano

**Affiliations:** 1 Department of Animal Science, University of California Davis, CA, 95616-8521, USA

## Abstract

**Background:**

Cow milk is a complex bioactive fluid consumed by humans beyond infancy. Even though the chemical and physical properties of cow milk are well characterized, very limited research has been done on characterizing the milk transcriptome. This study performs a comprehensive expression profiling of genes expressed in milk somatic cells of transition (day 15), peak (day 90) and late (day 250) lactation Holstein cows by RNA sequencing. Milk samples were collected from Holstein cows at 15, 90 and 250 days of lactation, and RNA was extracted from the pelleted milk cells. Gene expression analysis was conducted by Illumina RNA sequencing. Sequence reads were assembled and analyzed in CLC Genomics Workbench. Gene Ontology (GO) and pathway analysis were performed using the Blast2GO program and GeneGo application of MetaCore program.

**Results:**

A total of 16,892 genes were expressed in transition lactation, 19,094 genes were expressed in peak lactation and 18,070 genes were expressed in late lactation. Regardless of the lactation stage approximately 9,000 genes showed ubiquitous expression. Genes encoding caseins, whey proteins and enzymes in lactose synthesis pathway showed higher expression in early lactation. The majority of genes in the fat metabolism pathway had high expression in transition and peak lactation milk. Most of the genes encoding for endogenous proteases and enzymes in ubiquitin-proteasome pathway showed higher expression along the course of lactation.

**Conclusions:**

This is the first study to describe the comprehensive bovine milk transcriptome in Holstein cows. The results revealed that 69% of NCBI Btau 4.0 annotated genes are expressed in bovine milk somatic cells. Most of the genes were ubiquitously expressed in all three stages of lactation. However, a fraction of the milk transcriptome has genes devoted to specific functions unique to the lactation stage. This indicates the ability of milk somatic cells to adapt to different molecular functions according to the biological need of the animal. This study provides a valuable insight into the biology of lactation in the cow, as well as many avenues for future research on the bovine lactome.

## Background

Milk is a unique biological fluid consumed by mammalian infants. It contains many macro- and micro-nutrients that are essential for the growth and development of the newborn [1,2]. In addition, a diverse cocktail of bioactive factors, such as antibodies, oligosaccharides and nucleotides in milk, play immune, pre-biotic and protective functions in the infant gut [1,3,4].

Cow milk has an important role in human nutrition because cow milk-based infant formula is the most available substitute for human breast milk and cow milk is consumed beyond infancy in human populations around the world. However, there are significant differences between the physicochemical properties of human breast milk and cow milk [5]. Bovine milk contains a higher percentage of caseins whereas human milk has higher percentage of whey proteins, the highest being α-lactalbumin. β-lactoglobulin, the most abundant whey protein in bovine milk, is not present in human milk. These differences between human and cow milk in the amount and types of proteins have been suggested to be responsible for cow milk allergies in approximately 2-2.5% of human infants [6]. Oligosaccharides are abundant in human milk, and studies on milk demonstrate local and systemic beneficial effects to the suckling neonate [7,8]. The concentration of free oligosaccharides in bovine milk is reported to be approximately 20-fold lower than in human milk oligosaccharides [9]. The majority of these free oligosaccharides in bovine milk are sialylated whilst in human milk most of them are fucosylated [10]. Because of these differences between human and bovine milk, it would be desirable to change the composition of cow milk according to specific needs of target groups such as infants or immune compromised individuals. In order to achieve this goal, a thorough understanding of the components and the regulation of bovine milk composition is required.

Cow milk contains a heterogeneous population of somatic cells consisting of lymphocytes, neutrophils, macrophages and exfoliated epithelial cells [11]. These cells are responsible for the synthesis and secretion of components such as proteins, lipids and oligosaccharides in to the milk [12,13]. Even though many studies have been conducted on the physicochemical properties of cow milk and the genes expressed in bovine mammary gland [11,14], limited research has been published on the detailed characterization of genes expressed in somatic cells in milk. In a previous study we identified extensive similarities between the mammary gland and milk somatic cell transcriptome of the same cow [15]. Most of the genes expressed in the mammary gland transcriptome were present in milk somatic cells (MSC). Compared with the mammary gland, higher numbers of genes were expressed in MSC. Sets of genes related to immunity, organ development and behavior were uniquely expressed in MSC. Therefore, the identification and characterization of genes expressed in MSC represent an important step toward understanding the complex biological properties and species-specific variations of milk.

**Figure 1:**
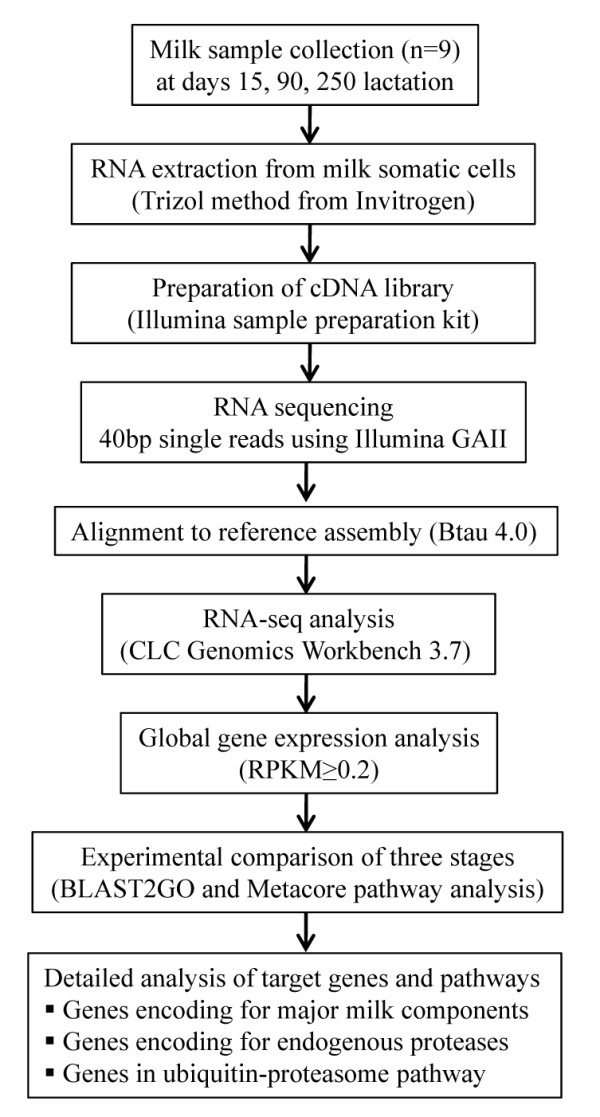
**Analytical flowchart to study bovine milk transcriptome by RNA-Seq**. Nine milk samples was collected at 15, 90 and 250 days of lactation with three biological replicates per stage. An initial global analysis was conducted on genes expressed in bovine MSC followed by a detailed analysis on genes encoding enzymes in major milk component synthesis pathways, endogenous milk proteases and enzymes in ubiqutin-proteasome pathway.

In the current study we used RNA sequencing (RNA-Seq) technology to examine the genes expressed in transition (day 15), peak (day 90) and late (day 250) lactation somatic cells in Holstein cow milk. Day 15 was selected to study the transition occurring from early lactation to peak lactation. Day 90 represents the peak lactation stage with the highest milk production. Day 250 represents the milk produced in the involuting mammary gland (in the initial stage). A global analysis was conducted first on the bovine milk transcriptome by studying the highly expressed genes in each stage of lactation and genes with statistically significant expression between the stages. Then a detailed analysis was conducted on the expression of important genes encoding enzymes in major milk component synthesis pathways (lactose, protein and fat), endogenous proteases and enzymes in ubiquitin- proteasome pathway. Figure 1 shows the analytical flow chart that was followed in the study.

## Results and Discussion

### Global analysis of gene expression in three stages of lactation

RNA sequencing produced a total of ~200 million reads with an average of 23 million reads for each stage of lactation. Three biological replicates were analyzed for each stage of lactation with the reads ranging from 11-28 million per sample. Approximately ~65% of the total reads uniquely mapped to the Btau 4.0 reference genome http://www.ncbi.nlm.nih.gov/genome/guide/cow/index.html. There were ~10% non-specifically mapped reads and 25% unmapped reads. Only the uniquely mapped reads were considered in the analysis. As described by in Bentley *et al. *[16] and Ramskold *et al. *[17] a threshold RPKM (reads per kilo base per million mapped reads) value was established to balance the number of false positives and false negatives. Detailed analysis of unique gene reads (number of sequence reads that match uniquely to the genes) and unique exon reads (number of sequence reads that match uniquely to the exons, including exon-exon junctions), revealed a threshold value of 0.2 RPKM for detectable gene expression in MSC [18]. For the genes with < 0.2 RPKM, a detailed analysis was conducted to identify the number of unique reads aligning outside the exonic regions due to annotation errors and the exclusion of new exons in the Btau 4.0 assembly. Using this strategy genes with RPKM values lower than 0.2 with more than 3 unique gene reads were also considered as genes expressed in MSC (Table 1).

**Table 1:** RNA-Seq gene expression results for three stages of lactation

Category^1^	Day 15	Day 90	Day 250
Highly expressed genes (≥ 500 RPKM)	86	140	150
Medium expressed genes (≥ 10 RPKM to 500 RPKM)	2850	5379	5038
Lowly expressed genes (< 10 RPKM)	13956	13575	12882

RPKM values ≥ 0.2	12083	13350	12610
RPKM values ≤ 0.2 with more than 3 unique gene reads	4809	5744	5460

Total expressed genes	16892	19094	18070

Non expressed genes	10476	8274	9298

^1^Genes were annotated using Btau 4.0 with total number of 27,368 genes

All the published gene expression studies conducted on mammary gland up to this point have used microarrays, and this is the first publication of RNA-Seq analysis of tissues related with lactation. Compared to microarray which is limited only to the probes on the array, RNA-Seq analysis considers all the genes expressed in a given tissue. A recent study conducted by Maningat *et al. *[19] on gene expression profiling of human milk fat globule using the human Ref-8 Illumina Bead-Chips with ~22,000 gene sequences, showed expression of 14,070 of these genes in human milk fat globule. This represents 63% of genes in the bead-chip. Considering that 14,070 genes were detected expressed in milk fat globule using 22,000 genes as reference, it was not unexpected that a higher number of expressed genes were detected by RNA-Seq in MSC.

In our analysis, 12,083 genes (44%) in transition (day 15) milk samples had RPKM values ≥ 0.2 and 4,809 genes (17%) had RPKM values ≤ 0.2 with more than 3 unique gene reads, therefore a total of 16,892 genes (61%) were considered as expressed in transition lactation MSC. In peak lactation (day 90), 13,350 genes (49%) had mean RPKM values ≥ 0.2 and 5,744 genes (20%) had RPKM values ≤ 0.2, therefore a total of 19,094 genes (69%) were considered as expressed in peak lactation. In late lactation (day 250) 12,610 genes (46%) had mean RPKM values ≥ 0.2 and 5,460 genes (20%) had RPKM values ≤ 0.2, therefore a total of 18,070 genes (66%) were considered as expressed in late lactation.

Peak lactation MSC had the highest number of expressed genes and transition lactation MSC had the lowest number of expressed genes. The 10 most highly expressed genes in transition lactation had very high RPKM values (3,039-265,630 RPKM), and they contributed to ~61% of the total gene reads. In peak lactation, the 10 most highly expressed genes (4,007-21,636 RPKM) contributed to ~11% of the total gene reads, and in late lactation, the 10 most highly expressed genes (3,011-31,398 RPKM) contributed to 19% of the total gene reads. Therefore, transition lactation milk showed the lowest complexity in the transcriptome with a smaller number of genes contributing to a larger fraction of the total mRNA while peak lactation milk showed the highest complexity.

### Analysis of genes with high expression in each stage of lactation

In order to categorize the genes with different level of expression, a multiphasic graph was obtained by plotting the log2 transformed RPKM values versus the expressed genes. According to the phases in the graph, gene expression RPKM values were categorized into three groups: high (≥ 500 RPKM), medium (≥ 10 to 500 RPKM) and low (< 10 RPKM) expressed genes. There were 86 (0.5%) highly expressed genes, 2,850 (17%) medium expressed genes and 13,956 genes (82.5%) with low expression in transition lactation MSC (Table 1). Genes with the highest RPKM values in transition lactation were *LGB *(β-lactoglobulin), *CSN2 *(β-casein), *CSN1S1 *(α-S1-casein), *LALBA *(α-lactalbumin), *CSN3 *(κ-casein), *GLYCAM1 *(glycosylation dependent cell adhesion molecule-1) and *CSN1S2 *(casein-α-S2) (Table 2). These genes showed a progressive decrease in expression in peak and late lactation. Except for *GLYCAM1 *the other six highly expressed genes encode caseins and whey proteins. *GLYCAM1*, a gene in the mucin family, encodes for a milk fat globule glycoprotein.

**Table 2:** Highly expressed genes in milk somatic cells at 15, 90 and 250 days of lactation

Category	Gene symbol^1^	Day 15^2^	Day 90^2^	Day 250^2^
Highly expressed genes in day 15	*LGB*	265631	12515	468
	*CSN2*	141267	13896	564
	*CSN1S1*	73573	11958	423
	*LALBA*	46445	4187	136
	*CSN3*	44747	5407	287
	*GLYCAM1*	25570	3415	124
	*CSN1S2*	20952	3116	129

Highly expressed genes in day 90	*SPP1*	2777	21637	9406
	*CSN2*	141267	13896	564
	*LGB*	265631	12515	468
	*CSN1S1*	73573	11958	423
	*FTH1*	3040	9798	15489
	*ACTB*	1212	6125	5830
	*CSN3*	44747	5407	287

Highly expressed genes in day 250	*FTH1*	3040	9798	15489
	*SPP1*	2777	21637	9406
	*CTSB*	1063	4463	6053
	*ACTB*	1212	6125	5830
	*APOE*	1099	1840	5315
	*CTSD*	786	4008	4646
	*CD74*	742	1951	4129

^1 ^Gene symbol according to NCBI Entrez gene database http://www.ncbi.nlm.nih.gov/gene/

^2^Days 15, 90 and 250 gene expressions given in RPKM

In peak lactation MSC there were 140 (0.7%) highly expressed genes, 5,379 (28%) medium expressed genes and 13,575 genes (71%) with low expression (Table 1). Genes with the highest RPKM values in peak lactation, were *SPP1 *(secreted phosphoprotein 1), *CSN2, LGB, CSN1S1, FTH1 *(ferritin heavy chain), *ACTB *(actin-β) and *CSN3 *(Table 2). The *SPP1 *gene encodes for osteopontin, a major phosphoprotein involved in bone remodeling [20]. Previous studies have shown strong associations between the *SPP1 *gene and bovine milk casein levels. An RNA interference study that depressed the expression of *SPP1 *gene showed decreased expression of *CSN2 *and *CSN3 *in a bovine mammosphere model [21]. It is thought that *SPP1 *enhances the expression of *CSN2 *and *CSN3 *by binding to integrin proteins on mammary epithelial cells [21]. Except for *FTH1*, the other six highly expressed genes in peak lactation MSC showed decreased expression in late lactation. *FTH1 *has shown increased expression during mammary gland involution in the mouse [22], and the progressive increase of *FTH1 *gene expression indicates an involvement of *FTH1 *in mammary gland involution in bovine. *FTH1 *has been identified as an anti-apoptotic factor [23], and it might be playing a protective role in the involuting bovine mammary gland. *ACTB *is a major component of the cytoskeleton involved in various types of cell motility functions. *ACTB *showed the highest expression in peak lactation MSC samples. The changes in expression of *ACTB *with the stages of lactation indicates that, even though *ACTB *is a popular housekeeping gene [24] that is used for gene expression normalization in many tissues, it cannot be used as a housekeeping gene in MSC over the course of lactation. This was also shown in an experiment conducted by Bionaz and Loor [25] in which they used pair-wise comparison of expression ratios along the course of lactation to predict the best housekeeping genes for the mammary gland.

Late lactation MSC had 150 genes (0.8%) with high expression, 5,038 (28%) genes with medium expression and 12,882 (71%) genes with low expression (Table 1). Genes with the highest RPKM values were *FTH1, SPP1, CTSB *(cathepsin B), *ACTB, APOE *(apolipoprotein), *CTSD *(cathepsin D) and *CD24 *(Table 2). There were remarkable differences between the highly expressed genes in late lactation MSC and genes expressed in transition and peak lactation MSC samples. Casein and whey protein genes were among the highly expressed genes in transition and peak lactation. However, late lactation showed high expression of a unique set of genes such as *CTSB *and *CTSD*. These genes belong to the cathepsins family, that are proteolytic enzymes found in many tissues, a discussion of genes encoding endogenous proteases in milk is presented later in the text. The *APOE *gene, which codes for Apolipoprotein-E, a lipid transporter with anti-apoptotic activity was also highly expressed [26]. A significant increase in *APOE *is observed along the course of lactation and, similar to *FTH1, APOE *may play an anti-apoptotic protective role in the involuting bovine mammary gland. *CD74 *is expressed in the B-lymphocytes, and the encoded protein promotes the proliferation of B-cells [27]. Therefore, unlike the other two stages of lactation, the highly expressed genes in late lactation MSC are involved mostly in proteolysis, anti-apoptotic activity and immune functions.

Previous studies conducted in our laboratory have shown many similarities between the genes expressed in mammary gland and MSC [15]. In the current study we have observed changes in expression patterns of different groups of genes in MSC along the course of lactation. Milk component related genes show higher expression in early lactation while the expression of apoptosis related genes increased toward the end of lactation. These suggest that the MSC are viable, transcriptionally active cells that can adapt to the conditions and needs of the lactation stage.

In order to examine the global functional changes associated with the expression of genes during lactation, all highly expressed genes (≥ 500 RPKM) at each stage of lactation were classified using the gene ontology (GO) terminology using the BLAST2GO software portal [28]. Highly expressed genes in three stages of lactation showed similar enrichment in most of the biological process, molecular function and cellular component GO terms. However, some GO terms showed differences in enrichment with the lactation stage. One important observation was the progressive increase in biological process GO terms for the immune process and biological regulation along the course of lactation (Additional File 1, Figure S1). Molecular function GO terms for electron carrier activity and proteosome regulator activity were detected only in peak and late lactation MSC samples. These results agree with previous gene expression studies conducted in the mouse mammary gland where immune related genes showed increased expression toward the later stages of lactation [29]. It is thought that immune cells are involved in defense, cytokine signaling and phagocytosis in the involuting mammary gland [29]. Increased immune cell function can lead to high protein turn over and this is reflected in enrichment of proteasome regulator GO term toward late lactation. Another important observation was that compared to the two other stages, the highly expressed genes in transition lactation MSC had a relatively high percentage of genes encoding proteins in non-membrane bound organelle locations.

Over all analysis of GO annotations showed that most of the genes expressed in MSC have similar functions and locations regardless of the lactation stage. However a fraction of the milk transcriptome has genes devoted to specific functions unique to the lactation stage, and proteins encoded by these genes function in specific cellular locations.

### Global analysis of genes with statistically significant changes in expression

Approximately 9,000 genes (53%) showed ubiquitous expression in bovine MSC. In order to identify the significant metabolic pathways represented by the genes with ubiquitous expression, a pathway analysis was conducted using the MetaCore portal (GeneGo, a Thomson Reuters business). Metabolic pathways represented by the genes with ubiquitous expression showed the involvement of most of these genes in housekeeping processes such as energy generation and cytoskeleton remodeling. The complete list of pathways and their p values are provided in the Additional File 2, Table S1.

The highest number of genes (6,243 genes) with significant expression differences (p ≤ 0.05, FDR q ≤ 0.3) was found between transition and peak lactation MSC. Among these genes 1,539 (24%) had higher expression in transition lactation MSC and 4,704 (76%) had higher expression in peak lactation MSC. BLAT2GO analysis conducted to study the functional changes associated with up regulated genes showed similar enrichment of GO terms in transition and late lactation. In agreement with observations in the previous section, the cells death biological process GO term and proteasome regulator molecular function GO term was detected only in peak lactation.

MetaCore pathway analysis of significantly up regulated genes at transition and peak lactation showed Phosphatidylinositol (3, 4, 5)-trisphosphate (PIP3) signaling pathway to have the most significant p value (p = 4.22 × 10^-5^) for enrichment in up-regulated genes in transition lactation MSC. PIP3 signaling activates the protein kinase Akt that regulates cell growth, proliferation and survival [30]. Lemay *et al *[31] observed the PI3-Akt pathway to be highly significant during lactation in mouse mammary gland. Therefore, our results agree with the observations of mouse mammary gland gene expressions, and the enrichment of PIP3 pathway in up regulated genes of transition lactation indicates an enhanced cell growth and proliferation in transition lactation compared to peak lactation. Cytoskeleton remodeling pathway had the most significant p value (p = 1.25 × 10^-13^) for enrichment for up regulated genes in peak lactation. However, the cytoskeleton remodeling pathway was also present in the 10 most significant pathways in transition lactation. There were several pathways that showed significant enrichment in both transition and peak MSC samples and this indicates that even though there are different sets of up regulated genes at two stages of lactation; most of these up regulated genes are involved in regulating the same metabolic pathways. The complete list of significant pathways and their p values are provided in the Additional File 3, Table S2.

There were 5,218 genes with statistically significant expression differences (p ≤ 0.05, FDR q ≤ 0.3) between transition and late lactation MSC. Among these genes, 1,257 (24%) had higher expression in transition lactation milk and 3,961 (76%) had higher expression in late lactation milk. BLAST2GO analysis showed similar enrichment of most of the GO terms in the up regulated genes at two stages of lactation. As expected, the cell death and proteasome regulator GO terms were only detected in genes up regulated in late lactation milk. MetaCore pathway analysis of these two groups showed cytoskeleton remodeling pathway to have the most significant p value (p = 9.58 × 10^-7^) for enrichment for up regulated genes in transition milk, and leukocyte chemotaxis pathway (p = 1.6 × 10^-12^) for up regulated genes in late lactation MSC. Late lactation showed significant enrichment of several pathways related to immune response and cell cycle. The complete list of significant pathways and their p values are provided in the Additional File 4, Table S3.

Peak and late lactation MSC showed more similarity in gene expression with only 238 genes with statistically significant expression differences (p ≤ 0.05, FDR q ≤ 0.3). Among these genes, 132 (55%) had higher expression in peak lactation and 106 (45%) had higher expression in late lactation. The BLAST2GO analysis of the up regulated genes showed similar enrichment of most of the GO terms. However, genes up regulated in late lactation had higher number of GO terms for cell proliferation, death, immune system process and growth (Additional File 5, Figure S2). The cell killing biological process GO term was detected only in genes with high expression in late lactation. Molecular function categorization showed significant high expression of genes with antioxidant activity in peak lactation. This shows that compared to peak lactation, somatic cells in late lactation milk were more involved in immune activity and involution process.

MetaCore pathway analysis showed statistically significant enrichment of G protein coupled receptors (GPRCs) involved in the regulation of smooth muscle contraction pathway in peak lactation MSC (p = 0.0003). Effect of the oxytocin hormone is mediated by the G-protein coupled receptors present in mammary epithelial cells [32] and milk secretion from lactating mammary gland is stimulated by the binding of oxytocin to these specific receptors [33]. High production and secretion of milk from the mammary gland in peak lactation can be the reason for an enrichment of GPRCs in the regulation of smooth muscle contraction pathway in peak lactation MSC. Role of the anaphase promoting complex (APC) in cell cycle regulation pathway had the most significant p value (p = 2.9 × 10^-5^) for enrichment in late lactation. Anaphase promoting complex is responsible for inducing the progression and exit of cell cycle from mitosis by APC induced proteolysis of different cell cycle regulators [34]. Enrichment of APC pathway in cell cycle regulation is a reflection of the biological changes occurring in the involuting mammary gland during late lactation. The complete list of significant pathways and their p values are provided in the Additional File 6, Table S4.

In summary, the blast2GO and the pathway enrichment analysis showed significant differences in the functions performed by genes with different expression in each stage of lactation. Genes involved in immune function, cell cycles and apoptosis showed significant higher expression towards the late lactation, where the mammary gland is in the initial stage of involution. This indicates that the MSC have the ability to adapt different molecular functions according to the biological need of the animal. However, we did not find enrichment of lactation specific pathways in any of the stages, most likely, these pathways are not well annotated in the gene ontology databases and pathways used for enrichment analysis.

### Expression of genes encoding for caseins, whey proteins and enzymes in lactose synthesis pathway

**Figure 2:**
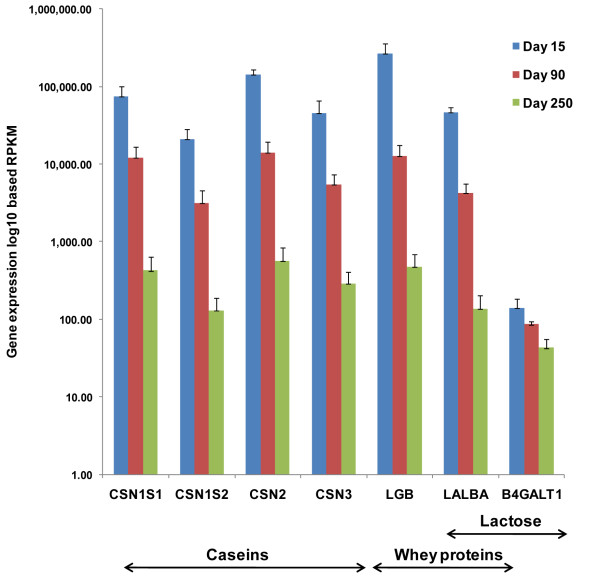
**Expression changes in genes encoding caseins, whey proteins and enzymes in lactose synthesis pathway along the course of lactation**. Gene symbols are shown in x-axis and the log2 RPKM expression values at 15, 90 and 250 days are shown in the y-axis. All the genes showed highest expression in day 15 and a significant decrease in expression with progression of lactation.

Caseins (CN) and whey proteins are the two major milk proteins in milk. Caseins are encoded by *CSN1S1, CSN1S2, CSN2 *and *CSN3*, a cluster of genes located on bovine chromosome 6. These genes encode the milk proteins α_s1_-CN, α_s2_-CN, β-CN and κ-CN, respectively [35]. All the CN genes showed very high expression in transition lactation MSC and a significant decrease in expression along the course of lactation. The *CSN2 *gene had the highest expression among the CN gene family throughout lactation, which agrees with the composition of caseins in which β-CN constitutes up to 45% of the caseins [36]. The main whey proteins, β-lactoglobulin and α-lactalbumin, are encoded by the *LGB *and *LALBA *genes, respectively. These two genes also showed very high expression in transition lactation and, similar to CN genes, a significant decrease in expression of whey protein genes during the course of lactation (Figure 2).

It is interesting that the percent of total milk proteins, whey proteins and casein along the course of lactation does not show a significant fluctuation along the lactation period [37]. However, the mRNA gene expression profiles in our study indicate higher transcription rate for casein and whey proteins in transition lactation. One possibility for this discrepancy is that these abundant caseins and whey proteins are broken in to bio-active peptides and therefore their concentration is not reflected in the analysis of major milk component proteins. It has been identified that the bio-active peptides formed by cleavage of caseins and whey proteins are higher toward the beginning of lactation [38,39]. One other possible explanation is that even though there is high expression of the genes encoding caseins and whey, the protein synthesis may not be efficient in transition lactation animals that are in negative energy balance or essential amino acids may be limiting.

STAT5 is the main transcription factor involved in inducing the expression of milk protein genes after hormonal induction. At least one binding site for STAT5 has been identified in milk protein genes [40]. Two STAT5 genes, *STAT5A *and *STAT5B*, are found in bovine, and both these genes showed higher expression in peak lactation MSC where the animals are in positive energy balance, and the milk yield is highest. Increased expression of *STAT5A *and *STA5B *in peak lactation may be a compensatory mechanism to maintain the milk protein composition during the rapid increase in milk yield.

Lactose is responsible for maintaining the osmotic pressure in milk. It is a unique product in the mammary alveolar cells and is produced by the enzymatic activity of lactose synthase, which is a combination of β-1, 4 galactosyl transferase encoded by *B4GALT1 *and α-lactalbumin encoded by *LALBA*. Both these genes showed higher expression in transition lactation and decreased expression during the course of lactation (Figure 2), and their expression patterns showed a positive correlation with the lactose concentration in milk, which decreases with advanced lactation [41].

### Expression of genes encoding enzymes in milk fat metabolism

**Figure 3:**
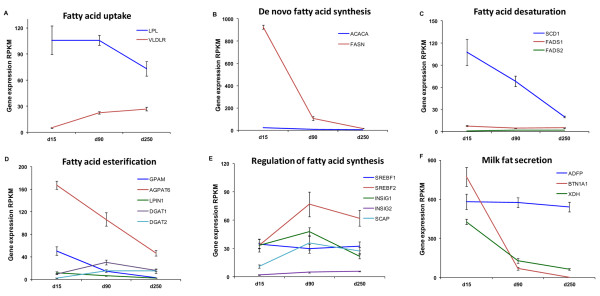
**Expression changes in genes involved in milk fat metabolism pathway along the course of lactation**. Stages of lactation are shown in x-axis and the RPKM gene expression values are shown in y-axis. Gene names are given in the legend. Fat metabolism in milk was divided in to six sections as: A) Fatty acid uptake, B) *De novo *fatty acid synthesis, C) Fatty acid desaturation, D) Fatty acid esterification, E) Regulation of fatty acid synthesis, F) Milk fat secretion. Most of the genes in fat metabolism pathway showed high expression in transition and peak lactation milk.

Milk fat metabolism in the mammary gland can be subdivided into five distinct processes: fatty acid uptake, *de novo *fatty acid synthesis, fatty acid desaturation, fatty acid esterification and milk fat secretion. All the important genes involved in these five processes were expressed in MSC and showed changes in their expression pattern along the course of lactation (Figure 3). Lipoprotein lipase (*LPL*) and very low density lipoprotein receptor (*VLDLR*) gene are two important genes involved in the fatty acid uptake pathway by mammary cells. *LPL *showed higher expression in transition and peak lactation, whereas the expression of *VLDLR *increased along the course of lactation (Figure 3A).

Acetyl-coenzyme A carboxylase alpha (*ACACA*), the gene encoding the rate limiting enzyme in *de novo *fatty acid synthesis, showed a significant decrease in expression along the course of lactation. A similar pattern of expression was observed with the fatty acid synthase (*FASN*) gene (Figure 3B). Compared to *ACACA*, the *FASN *gene showed higher expression throughout lactation.

Stearoyl-CoA desaturase (SCD1) is the main enzyme involved in mono-unsaturated fatty acid synthesis in ruminants [42]. SCD1 showed higher expression in transition lactation and decreased expression in peak and late lactation MSC. Enzymes for very long fatty acid desaturation is encoded by the fatty acid desaturase 1 and 2 (*FADS1, FADS*2) genes. Similar to previous observations in the bovine [25] and rat mammary gland [43], *FADS1 *showed a higher expression in MSC when compared to *FADS2*. Highest expression of *FADS1 *was observed in transition lactation, whereas *FADS2 *showed the highest expression in peak lactation (Figure 3C).

Glycerol-3-phosphate O-acyltransferase (*GPAM*), 1-acylglycerol-3-phosphate O-acyltransferase 6 (*AGPAT6*) and lipin 1 (*LIPIN1*) genes encode for the esterification enzymes in mono- and diacylglycerol synthesis. These three genes showed a similar pattern in expression with a high expression in transition lactation MSC and then a progressive decrease in expression along the lactation (Figure 3D). The *AGPAT6 *gene had the highest expression among these three genes throughout lactation. Diacylglycerol transferase 1 and 2 (*DGAT1, DGAT2*) genes encode for the major enzymes in TAG synthesis. Even though the expression of *DGAT2 *was low in transition lactation MSC, by the end of lactation the expression of *DGAT2 *was similar to that of *DGAT1 *(Figure 3D).

Sterol regulatory element-binding proteins 1 and 2 (*SREBF1, SREBF2*) play an important role in the transcription regulation of milk FA synthesis in the mouse [44] and cow [45]. *SREBF1 *showed a relatively constant expression along the course of lactation whereas *SREBF2 *had higher expression in peak and late lactation MSC (Figure 3E). Insulin induced 1 and 2 genes (*INSIG1, INSIG2*) and sterol-sensing proteins (SCAP) are involved in regulating *SREBF *activity. Both *INSIG1 *and *SCAP *had higher expression in peak lactation whereas *INSIG2 *had high expression in late lactation.

Butyrophilin (*BTN1A1*), Xanthine dehydrogenase (*XDH*) and adipophilin (*ADFP*) are main proteins involved in milk fat globule formation [25]. Genes encoding these proteins showed higher expression in transition lactation MSC and a significant decrease in the expression levels for *BTN1A1 *and *XDH1 *was observed during the course of lactation (Figure 3F). Expression levels for *ADFP *remained relatively constant along the course of lactation.

Most of the genes involved in lipid metabolism showed high expression in transition lactation MSC. *VLDLR *and *INSIG2 *had the highest expression in late lactation milk, whereas *FADS2, DGAT1, SREBF2, INSIG1 *and *SCAP *genes had highest expression in peak lactation MSC.

### Expression of endogenous proteases in bovine milk somatic cells

Important characteristics of dairy products, such as flavor, texture and longevity, are dependent mainly on proteins and fatty acids in milk [46]. Therefore, the endogenous proteases have an effect on the physicochemical characteristics of fresh milk and on the quality of dairy products such as yogurt and cheese [47]. Enzymatic activities of proteases such as plasmin [48], cathepsin D [49] and cathepsin B [50] are identified in the bovine milk by biochemical and immunological assays. However, no studies have been conducted on the expression level of the genes encoding these endogenous proteases.

The RNA-Seq expression analysis showed significant expression of cathepsin B, D, Z, H and S in the somatic cells of Holstein cow milk at days 15, 90 and 250 of lactation (Table 3). Except for cathepsin A and L1, all the other cathepsin genes showed an increase in expression during the course of lactation. This is the first time that expression of cathepsin genes has been measured in a mammalian milk sample, and the high expression of most of the cathepsin genes may indicate the presence of a high enzymatic activity of cathepsins in bovine milk.

**Table 3:** Expression values of genes encoding endogenous proteases in milk somatic cells at 15, 90 and 250 days of lactation

Category	Enzyme	Gene symbol^1^	Day 15^2^	Day 90^2^	Day 250^2^
Cathepsins	Cathepsin B	*CTSB*	1062.9	4463.3	6052.5
	Cathepsin D	*CTSD*	786.0	4007.6	4645.6
	Cathepsin Z	*CTSZ*	222.0	1048.3	1034.0
	Cathepsin H	*CTSH*	122.9	536.0	649.1
	Cathepsin S	*CTSS*	89.1	507.2	642.9
	Cathepsin C	*CTSC*	69.3	378.1	446.4
	Cathepsin K	*CTSK*	82.1	370.5	389.8
	Cathepsin A	*CTSA*	86.8	432.5	334.0
	Cathepsin F	*CTSF*	22.1	76.2	103.3
	Cathepsin W	*CTSW*	16.7	25.3	32.2
	Cathepsin L2	*CTSL2*	10.5	12.5	13.0
	Cathepsin O	*CTSO*	0.8	3.7	4.4
	Cathepsin L1	*CTSL1*	0.9	0.8	0.1
	Cathepsin G	*CTSG*	0.0	0.0	0.0

Elastase	Elastase 1	*ELA1*	0.4	3.2	1.2
	Elastase 3B	*ELA3B*	0.0	0.1	0.1

Plasminogen pathway	Plasminogen	*PLG*	0.0	0.0	0.0
	Urokinase-typeplasminogen activator	*PLAU*	18.7	216.8	52.2
	Urokinase receptor	*PLAUR*	60.4	346.1	91.3
	Tissue plasminogen activator	*PLAT*	1.9	4.2	4.8
	Plasminogen activator inhibitor	*SERPINE1*	3.6	79.7	19.5
	Plasmin inhibitor	*SERPINF2*	0.0	0.3	0.1

^1^Gene symbol according to NCBI Entrez gene database http://www.ncbi.nlm.nih.gov/gene/

^2^Days 15, 90 and 250 gene expressions given in RPKM

The plasminogen (*PLG*) gene was not expressed in any of the MSC samples. Previous studies have shown *PLG *as not being expressed in the bovine mammary gland [51], and the results of the present study confirm this also for MSC. The origin of plasmin and plasminogen is thought to be by migration from blood to the milk [52]. The lack of expression of *PLG *in MSC agrees with this migration theory of plasminogen. However, the other genes involved in the plasmin system were expressed in the MSC (Table 3). Urokinase type plasminogen activator (*PLAU*), urokinase receptor (*PLAUR*) and the plasminogen activator inhibitor (*SERPINE1*) had increased expression in peak lactation. These results indicate an active conversion of migrated plasminogen to plasmin in bovine milk and higher activity of plasmin in peak lactation milk. Even though elastase is a principal protease in neutrophils, there is no published literature on the enzymatic activity of elastase in milk. The elastase-1 gene showed expression in bovine MSC with the highest expression in peak lactation milk samples.

The gene expression analysis showed lower expression of proteases in transition lactation when compared to peak and late lactation MSC. Late lactation had the highest expression of cathepsins. Enzymes involved in the plasminogen activation pathway had high expression in peak lactation. Therefore, milk collected in earlier stages of lactation might have the minimum protease effect on the quality of dairy products. Plasmin in milk has been shown to cause bitterness in infant formula, and cathepsins have been shown to contribute to proteolysis of Swiss cheese [53]. With the present gene expression analysis results one can suggest that late lactation milk should be preferable to peak lactation milk for infant formula production and peak lactation milk may be optimal for Swiss cheese production. More research on biochemical and immunological assays on milk proteases are necessary to draw conclusions on selecting milk from the best lactation stage for the production of different dairy products.

### Expression of genes in ubiquitin- proteasome pathway

Ubiquitin-proteasome pathway (UPP) is a non-lysosomal, ATP-dependent pathway involved in degradation of proteins in the cell [54]. We have observed an enrichment of GO terms associated with UPP in peak and late lactation MSC. Lemay *et al. *[31] identified UPP as the most significantly enriched pathway during lactation and involution in mouse with the highest number of genes up regulated during early involution of the mammary gland. Because of the important regulatory functions performed by UPP, and the significant enrichment results observed in the current study and the mouse mammary gland, we analyzed the expression of 48 genes [54] belonging to UPP (Table 4).

**Table 4:** Expression values of genes encoding enzymes in ubiquitin-proteasome pathway at 15, 90 and 250 days of lactation

Protein	Gene symbol^1^	Day 15^2^	Day 90^2^	Day 250^2^
UB activating enzyme (E1)	*UBA1*	57.96	128.64	134.10

UB-conjugating enzyme (E2)	*CCDC34*	0.32	1.14	1.47
	*BRCA1*	0.48	1.87	2.71
	*UBE4B*	5.83	9.83	13.01
	*UBE4A*	8.19	23.34	17.83
	*UBE2R2*	10.91	21.70	26.34
	*UFD1L*	27.59	45.93	39.23
	*UBE2I*	14.54	37.27	42.38
	*UBE2C*	6.43	19.54	42.98
	*UBE2B*	16.53	48.69	43.34

UB-protein ligase (E3)	*SKP2*	0.24	1.00	1.07
	*CUL5*	0.93	1.80	2.39
	*ANAPC10*	1.63	3.12	3.05
	*SMURF2*	1.48	4.72	4.45
	*CUL4B*	3.02	4.87	5.29
	*CUL2*	1.62	6.14	5.96
	*BTRC*	2.11	12.92	6.21
	*SMAD3*	3.03	10.07	6.36
	*ANAPC5*	3.14	7.08	6.94
	*ANAPC1*	1.73	8.01	8.20
	*CUL3*	3.62	8.21	8.59
	*CUL1 *	4.88	14.32	14.64
	*FBXW2*	9.39	27.72	25.13
	*SMAD2 *	5.46	20.50	26.03
	*CUL4A*	15.25	59.93	44.91
	*SKP1*	14.72	61.88	48.03
	*STUB1*	42.41	55.86	52.47
	*RBX1*	33.24	42.01	76.38

UB and UB-like proteins	*SUMO1*	0.88	0.70	1.15
	*UBL7*	49.97	18.95	22.41
	*UBAP1*	8.78	32.96	28.78
	*NEDD8*	50.89	98.49	106.18
	*UBD*	32.48	163.89	259.41
	*UBC*	112.33	1095.10	332.69
	*RPS27A*	551.89	451.18	630.29
	*UBA52*	1710.22	1277.97	1903.08

Deubiquitination enzymes	*USP2*	0.60	0.37	0.23
	*USP9X*	0.05	0.46	0.56
	*USP21*	2.65	5.23	6.28
	*VCPIP1*	4.84	18.33	12.68
	*USP8*	4.54	10.15	14.05
	*USP10*	7.26	17.51	23.79

Proteasome	*ATAD2*	0.66	3.37	2.39
	*ATAD3A*	8.23	13.67	11.38
	*ATAD1*	4.43	9.99	13.15
	*PSMA1*	18.19	49.89	69.91
	*PSMD2*	41.15	82.81	112.20
	*PSMB2*	39.62	84.58	115.62

^1^Gene symbol according to NCBI Entrez gene database http://www.ncbi.nlm.nih.gov/gene/

^2^Days 15, 90 and 250 gene expressions given in RPKM

In UPP, degradation initiates by the covalent linkage of cellular proteins to multiple molecules of ubiquitin proteins (UB). Eight genes encoding UB and UB-like proteins (UBL) were expressed in bovine MSC. Among these genes, *UBA52 *had the highest expression at all three stages of lactation. *UBC *showed a significant high expression in peak lactation whilst most other UB genes showed higher expression in late lactation. Activity of three enzymes, UB-activating enzyme (E1), UB-conjugating enzyme (E2) and UB-protein ligase (E3) are required for the attachment of UB to the target proteins. Most the genes encoding these three enzymes had significantly higher expression in peak and late lactation. Once the target proteins are attached to UB there will be degradation by proteolytic activity in proteasomes. Six genes encoding proteasome proteins were expressed in the milk samples, and except for *ATAD3A*, all the others showed increased expression in late lactation. Deubiquitination enzymes regulate the overall proteolysis of UPP by removal of conjugated UB by proteolysis. Six deubiquitination enzymes were expressed in MSC with highest expression observed in *USP10 *at late lactation. All the genes, except *VCPIP1*, showed higher expression in late lactation. *VCPIP1 *had the highest expression in peak lactation.

The overall expression pattern of genes in UPP showed a higher expression of most of the genes along the course of lactation. This expression pattern agrees with pathway analysis results of the current study and the observation of Lemay *et al. *in early involuting mouse mammary gland [31] and it suggests a similar pattern of cellular protein degradation in mouse and cow with highest protein turn over occurring at later stages of lactation.

## Conclusions

This is the first published study on the global expression profiling of genes in the somatic cells of milk of any mammalian species. Sixty nine percent of genes annotated in NCBI Btau 4.0 bovine genome assembly were expressed in somatic cells. There was ubiquitous expression of ~9,000 genes while ~6,930 genes had a significant change in expression with the stage of lactation. The highest number of genes were expressed in peak lactation (day 90) MSC. Genes encoding caseins, whey proteins and enzymes in the lactose synthesis pathway showed high expression in transition lactation (day 15) MSC, and indicated higher production of casein and whey derived bio-active peptides. Most of the genes in fat metabolism also had high expression in transition and peak lactation MSC. There was an increase in the expression of genes in UPP along the course of lactation. Most of the endogenous milk proteases were expressed in peak and late lactation MSC and the important findings obtained from the detailed analysis of protease gene expression highlight the significance of metabolic pathway-based gene expression analysis. Analysis of the results obtained on all the gene network pathways are beyond the scope of this article and this is the first chapter of a fascinating journey on the biology of milk and milk somatic cells.

## Methods

### Animals and their management

Six healthy mastitis-free Holstein cows in their second or third lactation at the UC Davis dairy were selected for the study. The animals were kept in free stall housing, fed total mixed ration (TMR) and had access to water *ad libitum*. Cows were milked twice daily, at 4 a.m. and 4 p.m., in the milking parlor and managed according to AAALAC (American Association for Accreditation of Laboratory Animal Care) guidelines. Sample collection was approved by the UC Davis Institutional Animal Care and Use Committee (IACUC) protocol #16151. Microscopic examination with a trypan blue exclusion test for viability of milk somatic cells [55] was conducted in a preliminary study. Milk samples were collected from 4 cows (150-170 day lactation) at 0, 1, 2, 3 and 4 hours after the morning milking and examined under the microscope. The same procedure was repeated in another set of 4 cows in a separate day. The highest percentage of epithelial cells (11.6%) and the highest viability (86%) were detected 3 hours after morning milking. Therefore fresh milk samples were collected by hand milking the four quarters of the cows (50 ml from each quarter) at days 15, 90 and 250 of lactation 3 hours after the morning milking (three biological replicates per stage of lactation with a total of 9 samples). Initially the study was designed to analyze the milk obtained at days 15 and 250 and later, milk obtained at day 90 was included in the study. Therefore the day 15 and day 250 samples were collected from the same three cows whereas the day 90 samples were collected from three different cows. Day 90 cows were well matched to the other ones. Day 90 cows were selected from the same dairy and the animals were kept under the same management conditions. One animal collected at days 15 and 250 was in the third lactation, while the other two animals in these collection days were in their second lactation. Therefore, at day 90, one animal sample was collected from a cow in her third lactation and the other two samples were from cows in their second lactation.

### RNA extraction

Milk samples collected from the four quarters were mixed and a sub sample of 50 ml was used for RNA extraction from MSC. Milk cells were pelleted by adding 50 μl of 0.5 M EDTA to 50 ml of fresh milk and centrifuging at 1800 rpm at 4°C for 10 minutes. The pellet of cells was washed with 10 ml of PBS at pH7.2 and 0.5 mM EDTA and filtered through a sterile cheese cloth to remove any debris. Milk cells were then centrifuged again at 1800 rpm, 4°C for 10 minutes. The supernatant was decanted, and RNA was extracted from the milk cell pellet by the Trizol method (Invitrogen, Carlsbad, CA) according to the manufacturer's instructions. RNA was quantified by an ND-1000 spectrophotometer (Fisher Thermo, Wilmington, MA), and the quality and integrity was assessed by the spectrophotometer 260/280 ratio, gel electrophoresis and capillary electrophoresis with an Experion bio-analyzer (Bio-Rad, Hercules, CA).

### RNA sequencing and data analysis

Gene expression analysis (three samples per lactation stage) was conducted by Illumina RNA sequencing (RNA-Seq) technology. Messenger RNA was isolated and purified using an RNA-Seq sample preparation kit (Illumina, San Diego, CA). Then mRNA was fragmented to approximately 200 bp fragments and first and second strand cDNA were synthesized, followed by end repair and adapter ligation. The fragments were purified and sequenced at the UC Davis Genome Center DNA Technologies Core Facility using the Illumina Genome Analyzer (GAII). Short sequence reads of 36-40 bp were assembled and analyzed in RNA-Seq and expression analysis application of CLC Genomics Workbench 3.7 (CLC Bio, Aarhus, Denmark). The bovine genome Btau 4.0 http://www.ncbi.nlm.nih.gov/genome/guide/cow/index.html was utilized as the reference genome for the assembly. The following criteria were used to filter the unique sequence reads: minimum length fraction of 0.9; minimum similarity fraction of 0.8; maximum number of two mismatches. Data were normalized by calculating the reads per kilobase per million mapped reads (RPKM = total exon reads/mapped reads in millions × exon length in kb) [18] for each gene and annotated with NCBI bovine genome assembly (27,368 unique genes).

The experiment was carried out in two steps with day 15 and day 250 samples collected from the same cows and day 90 samples collected from different cows. Initial model checking was conducted to test for independence of samples collected at two time points from the same cow. Using R, a model was fit with animal and stage of lactation as two (non-interacting) factors on the expression of 27,368 unique genes in day 15 and day 250 samples. P values were obtained for the animal effect and the distribution of the p values was plotted. The majority of the genes had reasonably uniform p values between 0.4-0.5, and there was no significant animal effect on the analysis. Because of this uniform distribution of p values and the 235-day interval between samples collected from the same cow, samples were assumed to be independent of each other. T-tests and ANOVA were performed on log_2_-transformed data (0.5 was added to each number before log transformation to deal with zero counts) to identify the genes with significant changes in expression (p ≤ 0.05, FDR q ≤ 0.3) between the stages of lactations.

### GO annotation

Coding sequences of genes with high expression in each stage of lactation and genes that showed statistically significant changes between the lactation stages were obtained from the ENSEMBL biomart martview application http://www.ensembl.org/biomart/martview. These sequences were imported to the Blast2GO program to perform the blastx, mapping and GO annotation [28]. Statistical assessment of annotation differences between lactation stages were performed using all expressed genes as background and Fisher's Exact Test for multiple test correction in Blast2GO.

### Pathway analysis

MetaCore pathway analysis by GeneGo, a Thomson Reuters business, was used to identify the significant Gene GO pathways and Gene GO metabolic networks in genes with high expression and statistically significant changes in expression in each stage of lactation. This is calculated using a built in function of MetaCore software that uses a variation of the Fisher's exact test adjusted for multiple sample testing using the Benjamini-Hochberg FDR analysis. Human orthologs were used for the analysis.

## Authors' contributions

**SW **involved in design of study, sample collection, RNA-Seq experiments, acquisition, analysis and interpretation of data and drafting the manuscript. **GR **involved in design of study, sample collection, analysis and interpretation of data and revising the manuscript. **AI **involved in sample collection, RNA-Seq experiments and revising the manuscript critically. **JFM **involved in obtaining funds for the study, design of study, sample collection, interpretation of data and critically revising the manuscript. All authors read and approved the final manuscript.

## Supplementary Material

Additional file 1 **Figure S1: Biological process Blast2GO annotation results of highly expressed genes at three stages of lactation**. Biological process GO terms are shown in the x-axis. Percentages of genes belonging to each category are shown in y-axis. For all the three stages, the highest numbers of biological process GO-terms were found for cellular and metabolic process. Click here for file

Additional file 2 **Table S1**. Top GeneGo pathways identified in the genes with ubiquitous expression in MSC. Click here for file

Additional file 3 **Table S2**. Top GeneGo pathways identified in the genes with statistically significant changes in expression between transition lactation MSC and peak lactation MSC. Click here for file

Additional file 4 **Table S3**. Top GeneGo pathways identified in the genes with statistically significant changes in expression between transition lactation MSC and late lactation MSC. Click here for file

Additional file 5 **Figure S2: Comparison of biological process GO terms for the genes with statistically significant changes in expression between day 90 and day 250**. Biological process GO terms are shown in the x-axis. Percentages of genes belonging to each category are shown in y-axis. In both stages there is enrichment of cellular process and metabolic process GO terms. Day 250 milk had comparatively higher number of GO terms for cellular process, biological regulation, localization, signaling, cellular component organization, cell proliferation, death, immune system process, growth and multi-organism process. Click here for file

Additional file 6 **Table S4**. Top GeneGo pathways identified in the genes with statistically significant changes in expression between peak lactation MSC and late lactation MSC. Click here for file
