# Supplementary figures and images for: Transcriptional profiling of bovine milk using RNA sequencing
Source: BMC Genomics. 2012 Jan 25;13:45. doi: 10.1186/1471-2164-13-45 (PMC3285075; doi:10.1186/1471-2164-13-45)

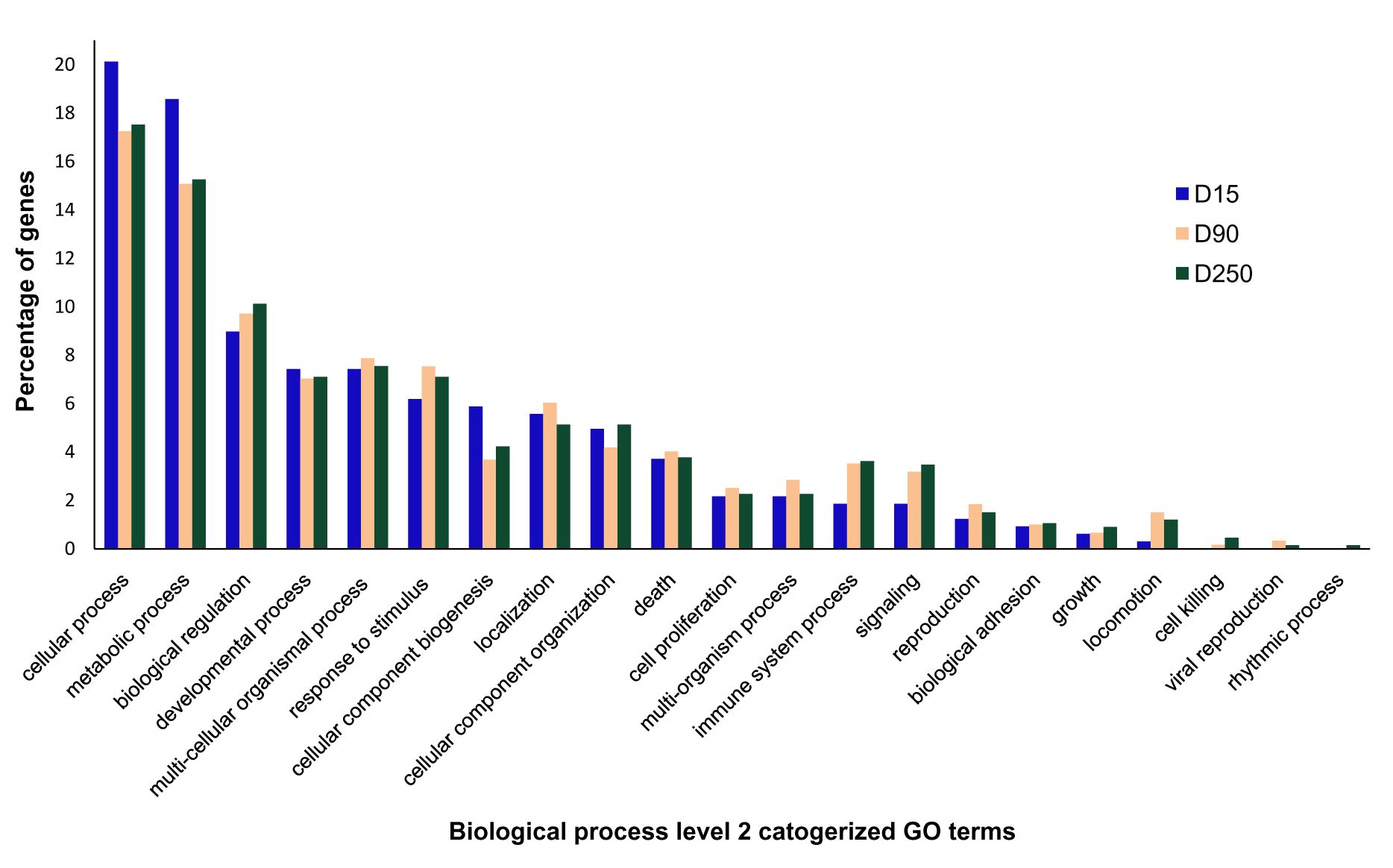

Supplement: Additional file 1 — Figure S1: Biological process Blast2GO annotation results of highly expressed genes at three stages of lactation. Biological process GO terms are shown in the x-axis. Percentages of genes belonging to each category are shown in y-axis. For all the three stages, the highest numbers of biological process GO-terms were found for cellular and metabolic process. [file 1471-2164-13-45-S1.TIFF]

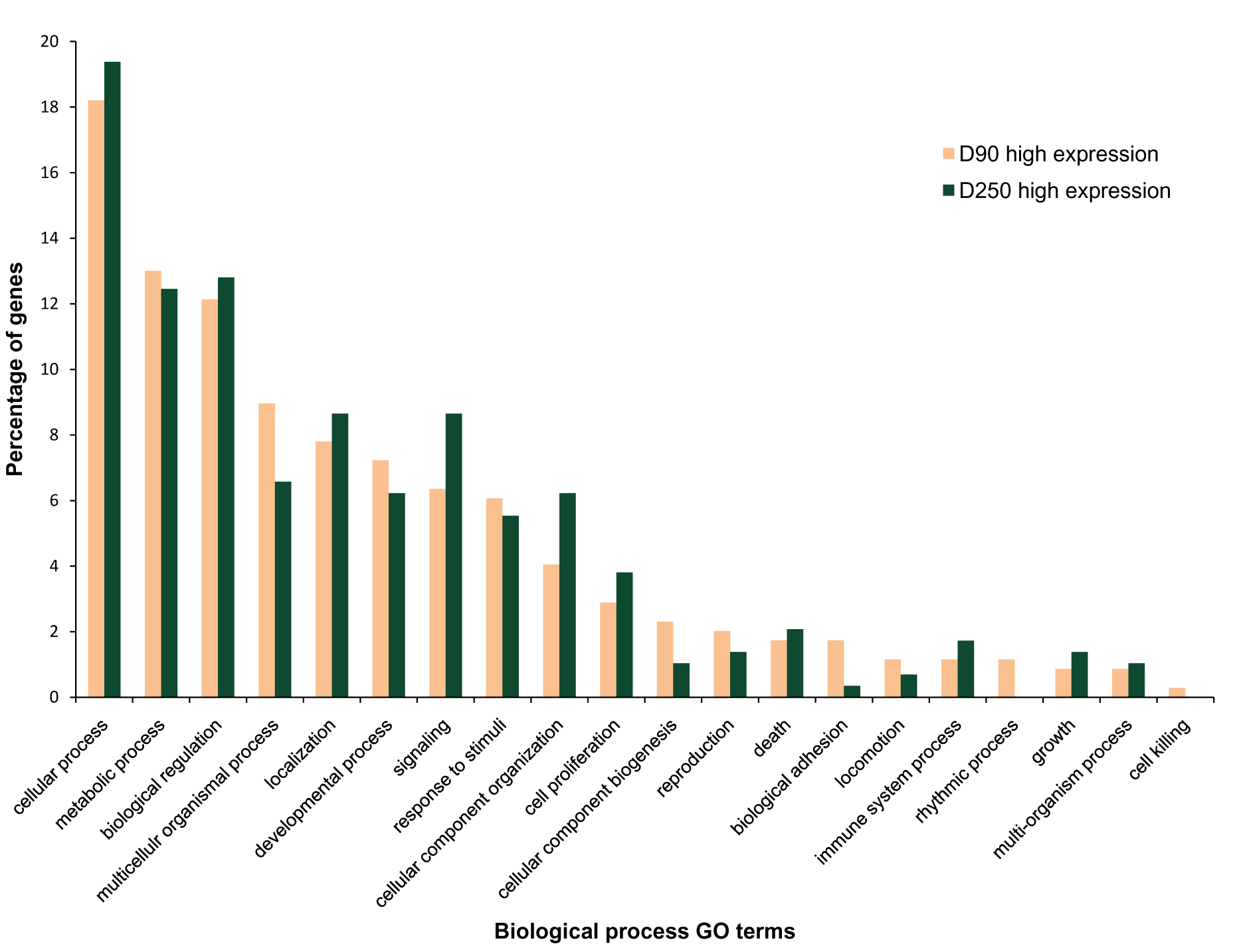

Supplement: Additional file 5 — Figure S2: Comparison of biological process GO terms for the genes with statistically significant changes in expression between day 90 and day 250. Biological process GO terms are shown in the x-axis. Percentages of genes belonging to each category are shown in y-axis. In both stages there is enrichment of cellular process and metabolic process GO terms. Day 250 milk had comparatively higher number of GO terms for cellular process, biological regulation, localization, signaling, cellular component organization, cell proliferation, death, immune system process, growth and multi-organism process. [file 1471-2164-13-45-S5.TIFF]
