# Supplementary material for: Transcriptional profiling of bovine milk using RNA sequencing
Source: BMC Genomics. 2012 Jan 25;13:45. doi: 10.1186/1471-2164-13-45 (PMC3285075; doi:10.1186/1471-2164-13-45)
Supplement: Additional file 2 — Table S1. Top GeneGo pathways identified in the genes with ubiquitous expression in MSC. [file 1471-2164-13-45-S2.DOC]

## Supplemental Table 1. Top GeneGo pathways identified in the genes with ubiquitous expression in milk

^a^Analysis was conducted by Gene Go pathway (about 650 signaling and metabolic maps) in Metacore program

| **Pathway name^a^** | **p value** |
| --- | --- |
| Oxidative phosphorylation | 1.34E-09 |
| Cytoskeleton remodeling_TGF, WNT and cytoskeletal remodeling | 1.58E-09 |
| Cytoskeleton remodeling_Neurofilaments | 3.71E-07 |
| Signal transduction_PKA signaling | 1.2E-06 |
| Cell adhesion_ECM remodeling | 2.02E-06 |
| Regulation of CFTR activity (norm and CF) | 2.67E-06 |
| Cytoskeleton remodeling_Keratin filaments | 7.28E-06 |
| Development_Role of IL-8 in angiogenesis | 9.33E-06 |
| Development_Regulation of epithelial-to-mesenchymal transition (EMT) | 1.06E-05 |
| Immune response_Histamine signaling in dendritic cells | 1.09E-05 |
