# Supplementary material for: Transcriptional profiling of bovine milk using RNA sequencing
Source: BMC Genomics. 2012 Jan 25;13:45. doi: 10.1186/1471-2164-13-45 (PMC3285075; doi:10.1186/1471-2164-13-45)
Supplement: Additional file 3 — Table S2. Top GeneGo pathways identified in the genes with statistically significant changes in expression between transition lactation MSC and peak lactation MSC. [file 1471-2164-13-45-S3.DOC]

## Supplemental Table 2. Top GeneGo pathways identified in the genes with statistical significant change in expression between transition lactation milk and mature lactation milk

| **Category** | **Name^a^** | **p value** |
| --- | --- | --- |
| Up regulated gene in day 15 | Development_PIP3 signaling in cardiac myocytes | 4.22305E-05 |
|  | Cytoskeleton remodeling_TGF, WNT and cytoskeletal remodeling | 4.96446E-05 |
|  | Cell cycle_Role of 14-3-3 proteins in cell cycle regulation | 0.000104645 |
|  | Cell cycle_Role of Nek in cell cycle regulation | 0.000126514 |
|  | Apoptosis and survival_DNA-damage-induced apoptosis | 0.000141866 |
|  | DNA damage_DNA-damage-induced responses | 0.000192586 |
|  | Cell cycle_The metaphase checkpoint | 0.000276659 |
|  | Cell adhesion_Cadherin-mediated cell adhesion | 0.000285282 |
|  | DNA damage_ATM / ATR regulation of G2 / M checkpoint | 0.000285282 |
|  | Cardiac Hypertrophy_NF-AT signaling in Cardiac Hypertrophy | 0.000555489 |
| Up regulated gene in day 90 | Cytoskeleton remodeling_Cytoskeleton remodeling | 1.25498E-13 |
|  | Cytoskeleton remodeling_TGF, WNT and cytoskeletal remodeling | 1.17617E-12 |
|  | Cell adhesion_Chemokines and adhesion | 5.8018E-12 |
|  | Immune response_IL-2 activation and signaling pathway | 6.0665E-12 |
|  | Immune response_CD28 signaling | 1.38293E-10 |
|  | G-protein signaling_Regulation of p38 and JNK signaling mediated by G-proteins | 2.00363E-10 |
|  | Immune response_Fc epsilon RI pathway | 2.42666E-10 |
|  | Immune response_CXCR4 signaling via second messenger | 3.70424E-10 |
|  | Cytoskeleton remodeling_Role of PKA in cytoskeleton reorganisation | 4.06382E-10 |
|  | Development_A1 receptor signaling | 4.99441E-10 |

^a^Analysis was conducted by Gene Go pathway (about 650 signaling and metabolic maps) in Metacore program
