# Supplementary material for: Transcriptional profiling of bovine milk using RNA sequencing
Source: BMC Genomics. 2012 Jan 25;13:45. doi: 10.1186/1471-2164-13-45 (PMC3285075; doi:10.1186/1471-2164-13-45)
Supplement: Additional file 4 — Table S3. Top GeneGo pathways identified in the genes with statistically significant changes in expression between transition lactation MSC and late lactation MSC. [file 1471-2164-13-45-S4.DOC]

## Supplemental Table 3. Top GeneGo pathways identified in the genes with statistical significant change in expression between transition lactation milk and late lactation milk

| **Category** | **Name^a^** | **p value** |
| --- | --- | --- |
| Up regulated gene in day 15 | Cytoskeleton remodeling_TGF, WNT and cytoskeletal remodeling | 9.57516E-07 |
|  | Cell cycle_The metaphase checkpoint | 2.2924E-06 |
|  | Cell adhesion_Cadherin-mediated cell adhesion | 1.87093E-05 |
|  | Development_PIP3 signaling in cardiac myocytes | 2.3865E-05 |
|  | Cell cycle_Role of 14-3-3 proteins in cell cycle regulation | 7.00135E-05 |
|  | Apoptosis and survival_DNA-damage-induced apoptosis | 0.000100741 |
|  | Role of alpha-6/beta-4 integrins in carcinoma progression | 0.000118696 |
|  | DNA damage_DNA-damage-induced responses | 0.000145508 |
|  | Cell adhesion_Plasmin signaling | 0.000146971 |
|  | Cytoskeleton remodeling_Cytoskeleton remodeling | 0.00016996 |
| Up regulated gene in day 250 | Chemotaxis_Leukocyte chemotaxis | 1.60969E-12 |
|  | Immune response _Immunological synapse formation | 3.90847E-11 |
|  | Blood coagulation_GPCRs in platelet aggregation | 2.86251E-10 |
|  | Cell cycle_Spindle assembly and chromosome separation | 8.12953E-10 |
|  | Cell cycle_Initiation of mitosis | 2.05046E-09 |
|  | Cell cycle_Start of DNA replication in early S phase | 3.8318E-09 |
|  | Cell cycle_Chromosome condensation in prometaphase | 9.91753E-09 |
|  | Cell adhesion_Integrin inside-out signaling | 1.24986E-08 |
|  | Blood coagulation_GPVI-dependent platelet activation | 4.40908E-08 |
|  | Cytoskeleton remodeling_Regulation of actin cytoskeleton by Rho GTPases | 5.46595E-08 |

^a^Analysis was conducted by Gene Go pathway (about 650 signaling and metabolic maps) in Metacore program
