# Supplementary material for: Transcriptional profiling of bovine milk using RNA sequencing
Source: BMC Genomics. 2012 Jan 25;13:45. doi: 10.1186/1471-2164-13-45 (PMC3285075; doi:10.1186/1471-2164-13-45)
Supplement: Additional file 6 — Table S4. Top GeneGo pathways identified in the genes with statistically significant changes in expression between peak lactation MSC and late lactation MSC. [file 1471-2164-13-45-S6.DOC]

## Supplemental Table 4. Top GeneGo pathways identified in the genes with statistical significant change in expression between mature lactation milk and late lactation milk

| **Category** | **Name^a^** | **p value** |
| --- | --- | --- |
| Up regulated gene in day 90 | Muscle contraction_ GPCRs in the regulation of smooth muscle tone | 0.000317458 |
|  | Cytoskeleton remodeling_Alpha-1A adrenergic receptor-dependent inhibition of PI3K | 0.002565292 |
|  | Glycine, serine, cysteine and threonine metabolism | 0.012612591 |
|  | Glycine, serine, cysteine and threonine metabolism/ Rodent version | 0.013176754 |
|  | Development_Activation of ERK by Alpha-1 adrenergic receptors | 0.013915908 |
|  | Translation_Translation regulation by Alpha-1 adrenergic receptors | 0.018987323 |
|  | GTP metabolism | 0.019668562 |
|  | Cell adhesion_Integrin inside-out signaling | 0.0210616 |
|  | Cardiac Hypertrophy_Ca(2+)-dependent NF-AT signaling in Cardiac Hypertrophy | 0.021773225 |
|  | Bacterial infections in CF airways | 0.022494804 |
| Up regulated gene in day 250 | Cell cycle_Role of APC in cell cycle regulation | 2.9074E-05 |
|  | DNA damage_ATM / ATR regulation of G2 / M checkpoint | 0.001125373 |
|  | Cell cycle_Role of Nek in cell cycle regulation | 0.001705828 |
|  | Cell cycle_Spindle assembly and chromosome separation | 0.00181382 |
|  | Cell cycle_The metaphase checkpoint | 0.002156858 |
|  | Reproduction_Progesterone-mediated oocyte maturation | 0.002658297 |
|  | Cell cycle_Nucleocytoplasmic transport of CDK/Cyclins | 0.026952214 |
|  | Apoptosis and survival_DNA-damage-induced apoptosis | 0.028851076 |
|  | Cell cycle_Cell cycle (generic schema) | 0.04017149 |
|  | Cell cycle_Chromosome condensation in prometaphase | 0.04017149 |

^a^Analysis was conducted by Gene Go pathway (about 650 signaling and metabolic maps) in Metacore program
